# Ubiquitination at pericentromeric regions: directing heterochromatin reassembly during cell division

**DOI:** 10.1038/s41392-026-02590-y

**Published:** 2026-02-20

**Authors:** Zhenxuan Chen, Tiebang Kang, Liwen Zhou

**Affiliations:** Sun Yat-sen University Cancer Center, State Key Laboratory of Oncology in South China, Guangdong Provincial Clinical Research Center for Cancer, Guangzhou, China

**Keywords:** Cell biology, Epigenetics

In a recent study published in *Nature*, Huang, Wong and their colleagues identified G2E3 as the major histone H3 lysine 14 mono-ubiquitin (H3K14ub) ligase in mammalian cells.^1^ By coupling G2E3-dependent H3K14 ubiquitination to the recruitment of SUV39H at pericentromeric chromatin, the authors define a conserved pathway that guides the reassembly of H3K9me3 domains after mitosis and secures the proper compartmentalization of chromatin.

H3K9me3-marked constitutive heterochromatin is essential for transcriptional repression and genome stability. In fission yeast, the CLRC complex mono-ubiquitinates H3K14 to stimulate the SUV39H homologue Clr4, providing an upstream ubiquitin signal for H3K9me3 deposition.^2^ Failure to re-establish H3K9me3 at constitutive heterochromatin destabilizes chromatin organization and potentially compromises chromosome segregation, raising the critical question of whether a similar H3K14ub-SUV39H mechanism operates in mammals. Huang and colleagues addressed this first by generating an H3K14ub-specific antibody, which was rigorously validated by dot blots and peptide competition assays, thereby establishing a robust foundation for the screen and genome-wide analysis. They then used the nuclear protein overexpression library to screen for factors increasing this mark. They identified the HECT-type ligase G2E3, which mono-ubiquitinates H3K14 in vitro and in cells. They further demonstrated that G2E3 is the principal writer of this modification, as mutation of the catalytic cysteine or substitution of H3K14 with arginine abolished H3K14ub, and G2E3 depletion markedly reduced global H3K14ub levels.

**Fig. 1:**
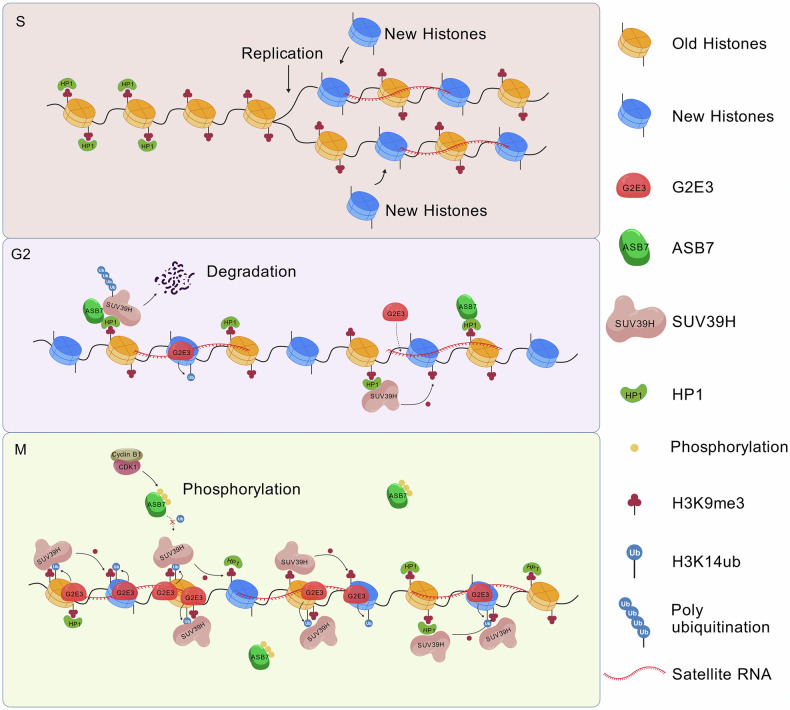
Ubiquitin ligase-mediated control of SUV39H dynamics safeguards H3K9me3 inheritance. During S phase (top), parental H3K9me3-marked histones are redistributed to both daughter strands, while newly synthesized histones lacking H3K9me3 are incorporated, leading to transient dilution of heterochromatin marks. In G2 (middle), ASB7 maintains SUV39H turnover, whereas G2E3 begins to accumulate and associate with satellite transcripts. Upon mitotic entry (bottom), CDK1-Cyclin B1 phosphorylates ASB7, temporarily suppressing its E3 ligase activity and allowing SUV39H to accumulate. G2E3, which is highly expressed in mitosis, is efficiently recruited to pericentromeric chromatin via satellite RNAs, catalyzes H3K14 ubiquitination, and facilitates robust recruitment of SUV39H to newly deposited nucleosomes. These coordinated ubiquitin-dependent mechanisms collectively ensure the faithful restoration of H3K9me3 at pericentromeric heterochromatin after DNA replication. This figure was created using BioGDP

Immunofluorescence analysis showed that H3K14ub is highly enriched in DAPI-dense pericentromeric heterochromatin, where G2E3 co-localizes with H3K9me3 and SUV39H. Loss of G2E3 substantially reduced H3K14ub foci, decreased pericentromeric H3K9me3, and impaired the re-binding of SUV39H2 and HP1 in late mitosis/anaphase. Biochemical assays further revealed that the chromodomain of SUV39H binds both H3K14ub and H3K9me3, with a marked preference for H3K14ub and the highest affinity for the dual-modified tail. These data support a model in which G2E3-mediated H3K14ub creates high-affinity docking sites that concentrate SUV39H at pericentromeric regions, thereby accelerating local H3K9me3 restoration Fig. 1.

Genome-wide profiling adds another aspect to this regulation. In wild-type cells, G2E3-dependent H3K14ub peaks coincide with H3K9me3 at pericentromeric satellite repeats and centromeric regions. G2E3 knockout reduces both marks at these loci but leads to widespread aberrant H3K9me3 accumulation in the euchromatic regions and probably transcriptional repression of the corresponding genes. In contrast, H3K9me3 at SETDB1-dependent repeats is largely preserved, suggesting that the G2E3-H3K14ub-SUV39H axis primarily controls pericentromeric heterochromatin and spatially restricts SUV39H activity.

This regulation is integrated with cell cycle control. G2E3 transcripts and H3K14ub peak in G2/M and direct SUV39H and HP1 to the heterochromatin after metaphase, then decline rapidly upon entry into G1. However, questions remain regarding how the cell cycle coordinates this sequential recruitment. Recent work on the CUL5 adaptor ASB7 showed that ASB7 promotes the degradation of SUV39H1 in S and G2 and is inactivated by CDK1-dependent phosphorylation during mitosis.^3^ Taken together, these findings suggest that ASB7 and G2E3 act in a functionally opposing, cell cycle-coupled manner, thereby fine-tuning SUV39H1 abundance and activity: during S/G2, active ASB7 constrains SUV39H1 abundance, whereas during metaphase, ASB7 inactivation coincides with maximal G2E3 activity to stabilize SUV39H and provide H3K14ub-mediated docking sites (Fig. 1). It will be important to determine whether the G2E3 protein is itself cell cycle regulated, whether it is a substrate of ASB7, and how these two E3 ligases jointly shape the kinetics of H3K9me3 recovery.

Another open question is how G2E3 recognizes pericentromeric regions. Huang and colleagues showed that G2E3 association with chromatin is sensitive to RNase treatment, and further RNA immunoprecipitation experiments indicated that G2E3 preferentially binds to α-satellite transcripts. These observations suggest that pericentromeric RNA contributes to G2E3 recruitment, but the underlying recognition mechanism is not yet defined. Future work will need to test whether G2E3 contains an intrinsic RNA-binding module or to identify RNA-binding proteins that may bridge G2E3 to satellite RNA. An additional question is whether ZNF512/512B are involved in directing G2E3 to chromatin, a possibility supported by recent findings showing that they recruit SUV39H to pericentromeric heterochromatin.^4^

A potential layer of regulation might involve mitotic phosphorylation. H3K14ub is detectable on metaphase chromosomes, whereas SUV39H and HP1 remain largely excluded from chromatin until late mitosis. This temporal separation suggests that H3K14ub is necessary but not sufficient for SUV39H engagement. One candidate modifier is H3S10 phosphorylation, which is abundant in mitosis and known to weaken HP1 binding to H3K9me3.^5^ It will be informative to examine how H3S10ph affects the interaction between the SUV39H chromodomain and H3K14ub-H3K9me3 using reconstituted nucleosomes with defined modification patterns and structures to test whether a phosphorylation-ubiquitination-methylation switch governs the chromatin rebinding of SUV39H and HP1.

Huang and colleagues also report that H3K14ub levels fall quickly as cells enter G1, indicating that this mark is actively removed once pericentromeric H3K9me3 domains have been re-established. However, the corresponding H3K14ub-removal mechanism remains unknown and may contribute to heterochromatin remodeling.

Finally, the conservation of H3K14ub-dependent H3K9me3 deposition from yeast CLRC/Clr4 to mammalian G2E3/SUV39H raises questions about genome stability and disease. By enabling rapid re-establishment of pericentromeric H3K9me3 and restricting SUV39H from euchromatin, G2E3 helps prevent abnormal chromosome segregation, aberrant satellite repeat expression, and genome instability. Although direct links to disease remain to be established, exploring whether G2E3 dysfunction contributes to tumor phenotypes associated with chromosomal instability or altered satellite transcription may have valuable translational implications.

Together, the findings of Huang, Wong and their colleagues establish G2E3-catalyzed H3K14 ubiquitination as a key upstream signal for pericentromeric H3K9me3 maintenance. They also outline a broader framework in which ubiquitin signaling, RNA and cell cycle regulation cooperate to preserve heterochromatin organization during cell division and, potentially, to modulate genome stability in disease.
